# Circulating microRNA Biomarkers as Liquid Biopsy for Cancer Patients: Pros and Cons of Current Assays

**DOI:** 10.3390/jcm4101890

**Published:** 2015-10-23

**Authors:** Shigeshi Ono, Stella Lam, Makoto Nagahara, Dave S. B. Hoon

**Affiliations:** Department of Molecular Oncology, John Wayne Cancer Institute, Providence Saint John’s Health Center, 2200 Santa Monica Blvd., Santa Monica, CA 90404, USA; E-Mails: onos@jwci.org (S.O.); lams@jwci.org (S.L.); Nagahara.srg2@tmd.ac.jp (M.N.)

**Keywords:** circulating microRNA, blood, cancer patients, diagnosis, prognosis, circulating nucleic acids, next-generation sequencing

## Abstract

An increasing number of studies have focused on circulating microRNAs (cmiRNA) in cancer patients’ blood for their potential as minimally-invasive biomarkers. Studies have reported the utility of assessing specific miRNAs in blood as diagnostic/prognostic biomarkers; however, the methodologies are not validated or standardized across laboratories. Unfortunately, there is often minimum limited overlap in techniques between results reported even in similar type studies on the same cancer. This hampers interpretation and reliability of cmiRNA as potential cancer biomarkers. Blood collection and processing, cmiRNA extractions, quality and quantity control of assays, defined patient population assessment, reproducibility, and reference standards all affect the cmiRNA assay results. To date, there is no reported definitive method to assess cmiRNAs. Therefore, appropriate and reliable methodologies are highly necessary in order for cmiRNAs to be used in regulated clinical diagnostic laboratories. In this review, we summarize the developments made over the past decade towards cmiRNA detection and discuss the pros and cons of the assays.

## 1. Introduction

MicroRNAs (miRNAs) are small, single-stranded non-coding RNA sequences of about 18–22 nucleotides that interact with specific target mRNAs [1,2,3,4,5]. They are known to have important roles at post-transcriptional and translational levels. It is estimated that miRNAs regulate approximately one third of the human protein-coding genome [6].

One of the first reports suggesting a role of miRNAs in cancer was published in 2002 [7]. Takamizawa *et al.* later demonstrated the prognostic value of miRNAs by showing that let-7 expression was decreased in lung cancer and the direct correlation between low let-7 expression levels and poor survival in lung cancer patients [8]. In 2005, Calin *et al.* reported the first study showing the diagnostic/prognostic importance of miRNAs at the genome-wide level [9]. Croce *et al.* reported that certain tumor-associated miRNAs were expressed by cancer-related regions, exhibiting DNA amplification, deletion or translocation during tumor growth [10]. These pioneer studies suggest the potential of miRNA expression utilized as biomarkers for cancer diagnosis and prognosis in tissues [11].

Current techniques for cancer diagnosis commonly require a biopsy of the cancer tissue. In addition to the invasive nature of this procedure, it is not always clinically feasible and is also associated with morbidity; thus, several studies have focused on the search for molecular circulating cell-free nucleic acids as cancer-biomarkers in human body fluids, such as in plasma and serum [12]. The field of circulating cell-free tumor DNA (ctDNA) in cancer patients has grown over the past two decades [13] and certain assays have entered the clinic as CLIA assays [14]. Circulating tumor cells (CTCs) have also been promising as blood biomarkers [15]. Weber *et al.* reported miRNAs were present in all of the 12 body fluids assessed, including plasma, urine, saliva, peritoneal fluid, pleural fluid, seminal fluid, tears, amniotic fluid, breast milk, bronchial lavage, cerebrospinal fluid, and colostrum [16], although Watson *et al.* later reported major concerns about these results [17]. Nevertheless, since discovering the existence of circulating miRNA (cmiRNA) in body fluids, the non-invasive “liquid biopsy” has been featured as a promising blood biomarker assay in various cancers. The notable stability and simple handling of cmiRNAs may make this a more suitable biomarkers-detection technique, compared to other molecular blood biomarkers, mainly due to its stability in room temperature [18,19,20]. Recently, Montani *et al.* have reported the value of cmiRNA for detecting early lung cancer [21], which suggests the utility of cmiRNAs for predicting not only disease prognosis but also screening of healthy individuals. Generally, miRNA levels are non-specific and associated with a wide range of conditions and outcomes. Unfortunately, there are few overlapping reports amongst the findings of relatively similar studies of the same cancer. Methodological inconsistency has been thought to be one of the reasons for this irregularity [22,23]. As of now, there is no robust, consistent, and accurate approach for measuring cmiRNA expression in plasma and serum, rendering its clinical application difficult (Table 1). Optimizing the standardization of cmiRNA is essential for the assays to be informative in the clinic for patient decision making.

In this review, we summarize the application as well as the pros and cons of various detection methods and the quantification of cmiRNAs.

**Table 1:** Examples of various methodologies for circulating microRNAs (cmiRNA).

Types of Cancer	Source	Anticoagulant	Volume (mL)	Isolation Method	Controls	Detection Method	References
Diffuse large B-cell lymphoma	Serum	N/A	2	TRIzol	miR-16	RT-qPCR	[24]
Prostate	Serum/Plasma	EDTA	10	mirVana PARIS	Cel-miRs	RT-qPCR pre-amp	[18]
NSCLC*	Serum/Plasma	Heparin	0.1	Total RNA purification kit	Cel-miRs	RT-qPCR	[25]
NSCLC*	Serum	N/A	0.5	mirVana PARIS	dCt matrix	RT-qPCR	[26]
NSCLC*	Serum	N/A	50	TRIzol	Normalization to total RNA	RT-qPCR, sequencing	[27]
NSCLC*	Plasma EV	U	3	Dynabeads mirVana PARIS	miR-142-3p,-30b	RT-qPCR	[28]
Lung	Plasma	EDTA	0.2	mirVana PARIS	RNU-6B	Microarray; RT-qPCR	[29]
HCC**	Plasma	U	0.25	miRNeasy	U6 snRNA; cel-miR-39	RT-qPCR TLDA cards A and B	[30]
Head and Neck	Plasma	EDTA	0.3	mirVana miRNA isolation kit	Cel-miR-39	TaqMan Array RT-qPCR	[31]
Gastric	Plasma	N/A	N/A	miRNeasy Mini kit	Cel-miR-39	RT-qPCR	[32]
HCC**	Plasma	N/A	N/A	N/A	miR-1228	RT-qPCR microarrays	[33]
RCC***	Serum	N/A	0.4	mirVana PARIS Kit	Cel-miR-39	RT-qPCR	[34]
Breast	Serum	N/A	N/A	N/A	miR-16	RT-qPCR-DS	[35]
Melanoma	Plasma	Sodium citrate	0.01	N/A	N/A	RT-qPCR-DP	[36]
Multiple myeloma	Serum	N/A	N/A	N/A	N/A	NanoString, RT-qPCR	[37]

* non-small cell lung cancer; ** hepatocellular carcinoma; *** renal cell carcinoma.

## 2. Blood Collection and Processing

Optimal conditions for collecting and processing blood specimens for cmiRNA assessment are yet to be determined. To prevent normal cell-derived miRNA contamination derived from the puncture site, discarding the first several ml of blood is important [38]. Blood must be processed within a few hours of collection to restrict contaminating levels of miRNA expression derived from lysed red blood cells, platelets, leukocytes, and circulating tumor cells in the cancer patients blood [39]. However, this is dependent on the type of blood collection tube used. Here we discuss the importance of utilization of appropriate blood collection tubes, which affects miRNA detection in both plasma and serum. Although previous approaches favored plasma for cmiRNA assessment, availability of newer types of blood collection tubes has made serum an alternative, albeit the more optimal fluid of the two remains a debatable topic. Nonetheless, serum contains more contaminating non-specific normal blood cell miRNA that may interfere with results’ specificity and interpretations.

Blood collected for cmiRNA analysis is usually processed as plasma or serum. The debate over which type is the best, remains ongoing, however, serum is known to have more non-specific cmiRNA due to the presence of cell-secreted clotting factors. Plasma is collected in tubes containing standard blood anticoagulants, including heparin, EDTA, or sodium citrate followed by centrifugation. Serum collection is derived from blood tubes without anticoagulants. Based on previous reports, there is little difference in miRNA quantification through plasma *vs* serum [18,40,41]. However, higher concentrations of some miRNA were found in serum [42], while higher levels of other miRNA were detected in plasma collected in EDTA-containing tubes [43]. This may be due to assay specificity and sensitivity issues. Recently, contaminating platelets, which contain a wide spectrum of miRNAs, are also considered to contaminate cmiRNA detection [44,45]. Moreover, anti-platelet therapy is reported to affect cmiRNA expression derived from platelets [46]. Together, these reports necessitate the development of standard protocols for blood specimen collection and processing, as well as disclosure of detailed patients’ clinical information in reports. Many of the discrepancies in results can be attributed to this early step in the process.

The duration and temperature conditions from the time of blood draw until the actual processing will influence miRNA levels. miRNA is more stable than DNA and mRNA, yet cryopreservation of plasma and serum must remain at −80 °C or below to prevent its potential degradation in long-term storage. Among the anticoagulant reagents for plasma, heparin is known to inhibit the reverse-transcriptase and polymerase enzymes used in PCR [47] and selectively affect the quantification of cmiRNAs in blood samples [48,49]. Heparinase treatment prior to reverse transcription quantitative-PCR (RT-qPCR) is effective, albeit its possible incomplete deactivation reduces RNA yield [50], therefore we believe the use of heparin must be avoided. Sodium citrate may also affect PCR result [51]; collection tubes containing EDTA were recommended over sodium citrate for miRNA assays by Fichtlschere *et al.* [52]; nonetheless, Kim *et al.* reported sodium citrate improved the sensitivity of miRNA detection compared with EDTA [50]. Currently, there is no single definitive reliable approach to processing blood for cmiRNA assays; to that end, detailed description of blood collection and processing methods in scientific publication must be reported. The Cell-Free DNA BCT^®^ (Streck, Omaha, NE, USA) plasma collector tubes for cfDNA such as in the FDA approved prenatal testing maybe optimal as they have been quite reliable for blood cfNA tests.

## 3. RNA Extraction Methods: Quantity and Quality Assessment of cmiRNA

### 3.1. RNA Extraction

Phenol-chloroform based methods, such as Trizol, which contains phenol and guanidinium thiocyanate, are sufficient [53]. Due to the small size of the miRNA molecules, overnight precipitation is necessary to efficiently recover the miRNA [45]. Small RNA molecules with low GC frequency are known to be selectively lost when using Trizol, especially when a small amount of blood was analyzed [54]. Currently most RNA and miRNA extractions are performed using a phenol-chloroform based extraction technique that requires a large sample volume [55,56]. One major existing issue in RNA extraction from blood is the formation of a large aqueous phase, caused by the addition of Trizol and the subsequent centrifugation. The amount of the aqueous phase is dependent on the ratio of Trizol to sample, but reducing the ratio will result in denaturation of proteins. In addition to the plasma or serum volume processed, this is the most inconsistent step reported in protocols. Unfortunately, most studies do not report the yield of cmiRNA recovered from each specific condition, which makes determining the efficiency of these extraction protocols difficult. The most significant obstacle to cmiRNA extraction is its small size, hence easily lost during the extraction and purification procedures.

Moreover, cmiRNAs are not only present in exosomes [57], but are also bound to blood proteins and lipids [35,36]; this creates a problem in interpreting total cmiRNA yields and depending on the isolation method utilized, can cause variabilities in the yield. cmiRNAs associated to exosomes can be found in microvesicles, whereas cmiRNAs bound to protein like Ago2 can be found in serum/plasma [57]. These cmiRNAs are protected from RNases in vesicles. Differential ultracentrifugation helps purify the different types of extracellular vesicles and ribonucleoprotein complex in serum/plasma [44,58]. But establishing the size and morphology requires other methods such as electron microscopy or size exclusion chromatography. It is suggested that a large portion of cmiRNAs are associated to protein bound complexes such as Ago2 which helps prevent degradation [57]. cmiRNAs in vesicles possibly have a function in cell-to-cell communication. Proteases and detergents are often employed to release bound cmiRNA [35]. The inconsistency of retrieval levels of cmiRNA from plasma and serum is problematic in regards to the amount of cmiRNA bound and must be carefully addressed. Thus, when reporting total cmiRNA one has to be careful of the extraction procedure and bound miRNA actually obtained. This is a problem and not yet resolved in the actual reporting of cmiRNA using various assays. True comparative analyses have not been well analyzed.

Recently, several miRNA extraction kits have become commercially available for research (Table 2). The recovery rate from total RNA isolation is dependent on the optimized procedures and volumes. Several manufacturers have utilized their own specific strategies and proprietary reagents for this purpose. MiRCURY™ RNA Isolation Kit (Exiqon, Denmark) which indicates miRNA can be isolated from biofluids, including blood; however, *mir*Vana™ PARIS™ (Life Technologies, Grand Island, NY, USA) and miRNeasy^®^ (Qiagen, Venlo, Limburg, Belgium) are more widely used for cmiRNA assays. Most studies do not mention the actual yield and quality of cmiRNA, which makes direct comparisons of these kits challenging. There are also several non-standard assays designed by individual laboratory groups and published, none have been validated. The accuracy of cmiRNA yields are important, since without it, identifying false negative results is virtually impossible. Therefore the yield of cmiRNA and quality need to be performed with accurate assays that are reproducible and robust. See below on various approaches to address this problem.

**Table 2:** Commercially available miRNA extraction kit.

Kit	Company	Sample Type	Remarks
mirVana™ PARIS™ Kit	Life technologies (Carlsbad, CA, USA)	Tissues, Cells	Protein can be isolated from the same sample
miRNeasy^®^ Mini Kit	QIAGEN (Venlo, Limburg, Blegium)	Tissues, Cells	
miRCURY™ RNA Isolation Kits	EXIQON (Vedbaek, Denmark)	*Biofluids*, Tissues, Cells, FFPE	Biofluids can be used as sources
mirPremier™ microRNA Isolation Kit	SIGMA-ALDRICH (St. Louis, MO, USA)	Tissues, Cells	No phenol and chloroform
miRNA Isolation Kit	FAVORGEN (Ping-Tung, Taiwan)	Tissues, Cells	No large RNA
MasterPure™ RNA Purification Kit	Epicenter (Madison, WI, USA)	Tissues, Cells	No spin column, No phenol and chloroform
microRNA Isolation Kit, Human Ago2	Wako (Osaka, Japan)	Tissues, Cells	IP* with human anti-Ago2 Ab
miRNA Purification & Isolation Kit	Takara/Clontech (Shiga, Japan)	Tissues, Cells	Protein can be isolated from the same sample.

Another existing challenge in clinical utility of cmiRNA is the sample size of both patients and healthy controls, which can invalidate assay result interpretations. A universal standardization of scientific data reporting is essential; by more clearly defining the parameters of the “Methods” section to implement particular requirements, such as the demographics details of the normal control samples to be compared, and the quantitation of the cmiRNA extracted. Scientific Journals can resolve the existing inconsistency in reporting and comparisons. Although many studies are reporting the presence of certain cmiRNA in cancer patients, it has been noted that several of these cmiRNAs are also elevated in healthy individuals and individuals with benign inflammatory diseases; since levels of cmiRNA vary based on gender, age, and health status (non-cancer), there has been much confusion in the literature that have reported particular cmiRNA as cancer blood biomarkers, although they are present in widely fluctuating levels in healthy individuals. The solution is to assess particular cmiRNAs used as cancer biomarkers in large normal control populations with well-defined representative demographics as mentioned above.

### 3.2. Quantity and Quality Assessment of miRNA

**Figure 1:**
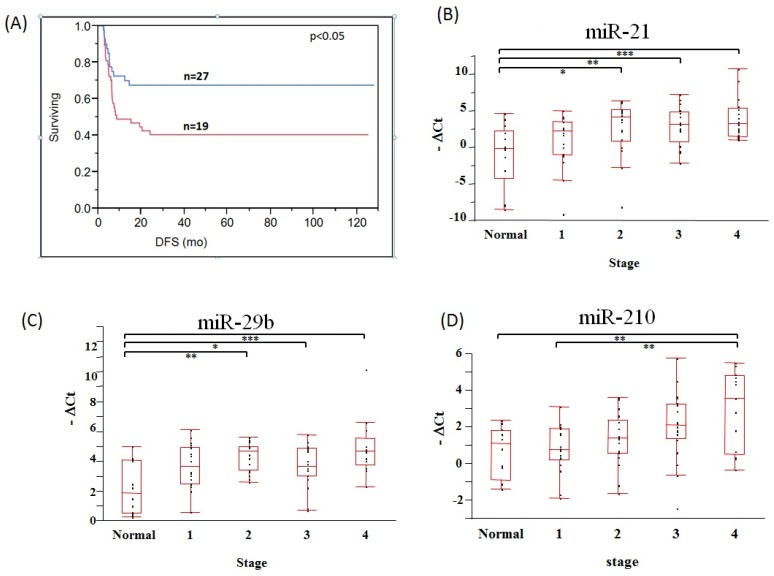
Direct cmiRNA assay of cancer patients (**A**) A level of miR-107 (50th percentile) in bleeds using direct cmRNA assay, taken at Day 0 significantly predict DFS. High levels predict worst prognosis; (**B**–**D**) Comparison of relative miRNA levels of breast cancer patients and normal samples in serum using a direct cmiRNA assay. The distribution chart shows each cmiRNA levels derived from normal samples *vs*. each AJCC stage.

There are several methods for assessing the quality and quantity of extracted RNA, including spectrophotometric analysis; however, determination of the ratio of miRNA to total RNA is challenging, since the absorbance for extraction solutions can interfere with assessing the nucleic acids. This may lead to an over estimation of cmiRNA quantity. Thus, it is difficult to distinguish mature miRNA from other small RNAs, including precursor miRNAs. In this aspect, several studies recommend using a fixed volume of serum/plasma, rather than a fixed miRNA amount for RT-qPCR [18,59]. Measurement of miRNA concentration is cumbersome, thus fixed amount of serum/plasma may be more efficient to assess the miRNA expression. Recently, we have demonstrated the efficacy of employing a small amount of serum and plasma for a direct (no extraction from serum/plasma) cmiRNA assay (<50 µL) [36,60]. Additionally, in our preliminary findings, we showed that miR-107 in stage III melanoma patients’ plasma is a biomarker for disease-free survival (DFS) (Figure 1A). We also assessed breast patients’ serum of different AJCC stages, and showed miR-21, miR-29b and miR-210 to increase during tumor progression (Figure 1B–D). These methods eliminate the potential loss of cmiRNA during the extraction procedure, and the need to consider the miRNA ratio to total RNA. This approach also provides a more robust way to analyze cmiRNA analysis and easier to perform in a clinical laboratory routinely. In addition to cmiRNA loss prevention, this direct assay proves to reduce the complexity and increase the efficiency of cmiRNA assessment [36].

As previously mentioned, quantitation of extracted cmiRNA can be difficult due to its low amounts. Agilent Technologies 2100 Bioanalyzer (Agilent, Santa Clara, CA, USA), which utilizes capillary electrophoresis, has been successful in assessing miRNA quantities [61]. This method provides RNA integrity number (RIN) to demonstrate miRNA quality; however, it still cannot discern precursor and mature miRNAs. A low RIN sample is considered not to be appropriate for microarray or NGS, but sufficient for RT-qPCR. RNA degradation is not as limiting for RT-qPCR as it is for NGSD and microarray analysis [62]. We consider a RIN below 8.0 to be too low for next-generation sequencing (NGS). To assess cfNA by NGS, one must perform deep sequencing to adequately assess majority of the miRNA, otherwise the sequencing results will be variable and not often representative of all cmiRNA. Currently employed traditional approaches of cmiRNA analysis by NGS are not very informative.

## 4. Methodological Variations of cmiRNA Detection Profiling

Currently, several methods have emerged to examine cmiRNA levels including RT-qPCR, microarrays and NGS. Each method has its pros and cons ranging from simplicity, quantification, and validity (Table 3). The sensitivity and specificity derived from these methods is often dependent on the type of samples and volumes of plasma or serum.

**Table 3:** Pros and cons of methodological variations.

	Sensitivity	Specificity	Accuracy	Analysis	Reproducibility	Discovery
RT-qPCR	++++	++++	++++	Easy	++++	Impossible
Affymetrix GeneChip miRNA Arrays 4.0	+	+	+	Moderate	+	Impossible
Agilent oligonucleotides microarrays	+	+	+	Moderate	+	Impossible
Exiqon miRCURY LNA microRNA arrays	++	++	++	Moderate	+	Impossible
µParaflo^®^Microfluidic Biochip Technology	+	+	+	Moderate	+	Impossible
3D-Gene^®^	+++	+++	+++	Moderate	++	Impossible
Next-generation sequencing	++	++	++	Difficult	+	Possible

Low to high: + to ++++. Utility scale.

### 4.1. RT-qPCR

Both the TaqMan^®^ and SYBR^®^ Green RT-qPCR assays are capable of analyzing the cmiRNA expression successfully. Each assay has specific reagents and protocols and is compatible with various PCR thermocyclers, thus introducing different quantitative and qualitative cmiRNA analysis.

Relative (comparative *Ct*) RT-qPCR is often used for cmiRNA analysis to measure the changes in gene expression of each sample to a suitable internal control. As of now, several internal controls have been used, including hsa-miR-16, hsa-miR-30b, and hsa-miR-142-3p, as well as the small RNA U6 and RNU-6B [24,28,29,30,35], though none of them are globally standard. For example, hsa-miR-16 has been most widely used as an internal control, but now it is known to be varied in several diseases and normal individuals [32,63,64,65]. Moreover, small RNA species such as RNU-6B is not native to human serum/plasma and is known to degrade during storage [66]. In addition, they are transcribed from a different RNA polymerase and may have different functions than from miRNA. This is problematic due to its presence in cancer patients, as well as normal individuals. Depending on the type of assay used, the resulting information may be false. U6 is recently reported to be an unsuitable internal control [67]. The stability of U6 expression is found to be less in serum especially after a number of freeze-thaw cycles. Some studies have suggested an external control to normalize the level of circulating miRNAs. The exogenous references are non-human mature miRNAs, including cel-miR-39, cel-miR-54, and cel-miR-238 [18,43,52,68]. These spike-in external controls are recommended as a measure of quality control for the RNA extraction and possibly RNA samples. However, it is difficult to control the amount of this artificial external control added into different samples. The artificial miRNAs are reconstituted in molecular biology grade or nuclease-free water at a set concentration followed by serial dilutions and stored in −80 °C. These artificial miRNAs are spiked-in to the samples at the lysis buffer step prior to RNA extraction. Precautions must be taken when adding these non-human external controls, because severe contamination can occur in samples. Baggish *et al.* used synthetic hsa-miR-422b, as it is minimally expressed in plasma [69]. As of now, we think exogenous references would be most useful, nevertheless further studies are needed to identify miRNAs that can serve as true universal miRNA controls. This is a major flaw of most assays reported. Standardization for cmiRNA quantification must be developed as for mRNA using similar approaches of MIQE guidelines [70,71]. Reproducible and comparable assay quantification are also issues in cfDNA analyses to date. Future cooperative studies are needed to define the parameters of cmiRNA quantification and reproducibility of assays reported.

On the other hand, absolute (standard curve) RT-qPCR may be used for analytical measurements of miRNA present in a given sample. One approach requires generating a standard curve for each miRNA, which is quite costly due to the amount of time and labor it requires. Furthermore, it is crucial that the stock sample used in generating these curves, to be accurately diluted each time with sufficient quantity to run multiple assays. The stability of the diluted standard curves must also be considered in regards to proper storage and freeze-thaw events prior to use. Alternatively, Droplet digital PCR (ddPCR) does not require a reference standard curve or an endogenous control. Instead the samples are divided and a ratio of positive (target molecule) to negative (no target) is used to count the number of target molecules in the sample, to allow accurate detection of low copy or rare allelic amplification. A study shows the potential use of ddPCR in miRNAs quantification and in this case in sputum for lung cancer diagnosis [72]. Additionally, pre-amplification may be necessary at times especially with low input sample or sample with low concentrations. However, it is important to consider that pre-amplification of the target samples may affect the PCR amplification and potentially produce bias in ddPCR results. The most significant drawback is the consumable costs and instrumental degree of specificity associated with ddPCR. There are different systems and instrument using ddPCR whereby, each have different sensitivities.

### 4.2. Microarray

Recently, microarray-based assays have also been widely applied to detect expression profiles of cmiRNAs (Table 4). The advantage of the microarray approach is its ability to assess genome-wide profiling of large numbers of cmiRNAs in blood and to identify candidate biomarkers for diagnostic and prognostic purposes in cancer patients. However, specific imaging systems and data analysis software are required to perform these methodologies. Depending on the manufacturers, they differ according to the reagents related to miRNA labeling, as well as methods and probe design used to immobilize the probes [22,73]. Direct and indirect miRNA labeling methods have been reported as follows [22,74,75]. For direct methods, T4 RNA ligase is used to directly add a fluorescent-modified nucleotide on the 3′-terminal of the miRNA. Another direct labeling method involves Poly-A tailing of the 3′-terminal. The latter overcomes the problem of circularization but might add various nucleotides in the tailing step, potentially altering hybridization properties [22]. On the other hand, for indirect labeling methods, RT is performed with amine-labeled dNTP mix, and the cDNA products are subsequently labeled with fluorescent dyes [75].

**Table 4:** Summary of microarrays for cmiRNA.

Assay	Required Input (ng)	Probe Content
Affymetrix GeneChip miRNA Arrays 4.0	130	miRBase v.20
Agilent oligonucleotides microarrays	100	miRBase v.21
Exiqon miRCURY LNA microRNA arrays	30	miRBase v.19
µParaflo^®^Microfluidic Biochip Technology	1000	miRBase v.21
3D-Gene^®^	2000	miRBase v.21

As opposed to RT-qPCR, microarrays cannot be used for absolute quantification due to their lower sensitivity and specificity compared to RT-qPCR [22]. Moreover, arrays require a larger amount of total RNA and a pre-amplification step, which introduces risks of changing the original concentration of the cmiRNAs. Mestdagh *et al.* systematically compared 12 commercially available platforms for analysis of miRNA expression and determined each methods strengths and weaknesses [76]. They evaluated rates of miRNA detection in serum samples and determined RT-qPCR platforms provided higher sensitivity, accuracy, and reproducibility compared to microarray or sequencing platforms. They also concluded appropriate platforms should be chosen on the basis of the experimental setting. Chen *et al.* also quantified cmiRNA expression using both RT-qPCR and microarray and noted a weak correlation, implying the possibility of inaccuracies when using microarray-based methods [77,78]. In general microarray platforms for cmiRNA have not been very robust and have limited sensitivities as compared to PCR based assays. Recently, a highly sensitive 3D-Gene^®^ (Toray, Tokyo, Japan) microarray was developed and reported in several publications [79,80,81]. It is not only sensitive but also has a high reproducibility that may contribute to the utility improvement of cmiRNA analysis.

### 4.3. Next-Generation Sequencing

Massive parallel sequencing (MPS) has been thought to be a current and promising technology for miRNA biomarker discovery. Knowledge of target miRNA and specific probes or primers is not necessary for this analysis, which enables investigators to assess unknown miRNAs.

In sample preparation, after total RNA extraction is followed by size fractionation of the small RNA population, RNAs are converted to cDNA. Adapter ligation and PCR amplification of cDNA is then performed according to the library preparation method appropriate for the respective MPS platform.

miRNA sequencing with library kits such as Illumina TruSeq Small RNA kit allows for direct sequencing and quantification of miRNA in samples and even extremely low expressing miRNAs can be detected. However, size selection is a tedious process, prone to human error and batch effect; library construction is also time consuming and requires a high input of high quality RNA.

Recently, the HTG EdgeSeq system (HTG molecular, Tuscon, AZ, USA) has developed a new approach of cmiRNA. This approach simplifies sample preparation for targeted sequencing of >2000 miRNAs [82]. This system does not require RNA extraction or manual library construction, and the fast and simplified protocol is highly automated with less user-related variation, reduced sample preparation and input requirements, and allows for detection of extremely low expressing miRNAs. The HTG EdgeSeq system relies on the specificity of the pre-designed probes and the *S1* enzyme digestion. Further validation is ongoing to determine its specificity and sensitivity in detecting cmiRNA.

As with all NGS assays, data analysis requires specific miRNA bioinformatics support. In addition, relative miRNA quantification is dependent on the sequencing read depth and appropriate normalization of the sequence reads. Other disadvantages of MPS assays are the required time and cost; MPS takes 1 week per run including sample preparation, which is longer compared to RT-qPCR. Although the cost is decreasing, it is still higher in comparison to RT-qPCR assays. However, the cost of individual miRNA detection is yet higher in PCR *vs*. microarray or NGS, implicating that an appropriate strategy must be carefully considered for each study design.

## 5. Discussion

Much progress has been made in methodological approach of cmiRNA detection profiling. However, given the significance of quality control in RT-qPCR microarray and MGS, the quality and quantity of the cmiRNA strongly affects the detection level of analysis. Traditional phenol-chloroform based RNA extraction techniques and several extraction kits are available; nevertheless, there is no gold standard for assessing cmiRNA. Since quality control is a major step in miRNA analysis prospective studies are highly necessary to reach a consensus on this important issue.

In discussing the pros and cons of RT-qPCR, microarray, and NGS, we must compare the complexity, throughput, sensitivity/specificity, necessary time, required RNA input, and associated costs among them. RT-qPCR is the most useful for assessing several known specific miRNA because it is easily and quickly performed, in addition to having the highest sensitivity and quantification. Microarray and NGS are used for high-throughput or unknown targets, but accuracy, and cost have been a problem.

Despite the recent reduction in the cost of microarrays and NGS, and their improved computational accuracy, RT-qPCR remains the most widely used method in validating microarray and NGS results, likely because it exhibits the highest relative sensitivity and specificity. The search for useful diagnostic and prognostic cancer biomarkers obtained from “liquid biopsy” is in high demand. As highlighted throughout this review, employing microarrays and NGS for discovering novel cmiRNAs is quite promising. In addition, careful validation needs to be performed using RT-qPCR. In this phase, next crucial step is to define a robust standard methodology, including an endogenous control. While an abundance of studies report differential detection of miRNAs, the important procedural details have not been provided. Large scale, inter-laboratory reproducibility and assessment must be facilitated through methodological standardization. Many assays are available for tissue miRNA evaluation; however, adaptation to cmiRNA is not easily adaptable and reproducible. It is clear that more effort is needed in isolating and assessing cmiRNA more efficiently. Similar limitations exist in the analysis of circulating cell-free DNA in cancer patients.

cmiRNA biomarkers as liquid biopsy is most promising not only for cancer patients but also healthy individuals with benign diseases. Cancer screening, staging, and response to treatment may be assessed by evaluating specific miRNA expression levels in body fluids. As previously discussed, the technology of cmiRNA extraction and profiling has improved considerably. Translating basic molecular research into clinical biomarkers of relevance, calls for prospective multicenter studies to validate specific cmiRNAs using verified extraction and assay methodologies that have standardization qualities built in.

## 6. Conclusions

The methodology of assessing cmiRNAs still lacks consistency and standardization, which is causing discrepancies between the studies reported. Further efforts are required to establish standard result-reporting parameters for comparison verification of individual cmiRNA. Assessment of cmiRNAs as biomarkers has compelling potentials owed to their inherent properties. By developing more efficient assays, their clinical utility in cancer patients will be better demonstrated.
