# Correction: Ono, S.; Lam, S.; Nagahara, M.; Hoon, D.S.B. Circulating microRNA Biomarkers as Liquid Biopsy for Cancer Patients: Pros and Cons of Current Assays. *J. Clin. Med*. 2015, *4*, 1890–1907

**DOI:** 10.3390/jcm5090081

**Published:** 2016-09-12

**Authors:** Shigeshi Ono, Stella Lam, Makoto Nagahara, Dave S. B. Hoon

**Affiliations:** Department of Molecular Oncology, John Wayne Cancer Institute, Providence Saint John’s Health Center, 2200 Santa Monica Blvd., Santa Monica, CA 90404, USA; onos@jwci.org (S.O.); lams@jwci.org (S.L.); Nagahara.srg2@tmd.ac.jp (M.N.)

The authors wish to make the following corrections to this paper [1]:

**Table 4:** Summary of microarrays for cmiRNA.

Assay	Required Input (ng)	Probe Content
Affymetrix GeneChip miRNA Arrays 4.0	130	miRBase v.20
Agilent oligonucleotides microarrays	100	miRBase v.21
Exiqon miRCURY LNA microRNA arrays	30	miRBase v.19
µParaflo^®^Microfluidic Biochip Technology	1000	miRBase v.21
3D-Gene^®^	2000	miRBase v.21

should be replaced with

**Table 4:** Summary of microarrays for cmiRNA.

Assay	Required Input (ng)	Probe Content
Affymetrix GeneChip miRNA Arrays 4.0	130	miRBase v.20
Agilent oligonucleotides microarrays	100	miRBase v.21
Exiqon miRCURY LNA microRNA arrays	30	miRBase v.19
µParaflo^®^Microfluidic Biochip Technology	1000	miRBase v.21
3D-Gene^®^	250	miRBase v.21

The authors apologize for any inconvenience caused to the readers by these changes.
